# A Physiologically-Based Pharmacokinetic Framework for Prediction of Drug Exposure in Malnourished Children

**DOI:** 10.3390/pharmaceutics13020204

**Published:** 2021-02-02

**Authors:** Erik Sjögren, Joel Tarning, Karen I. Barnes, E. Niclas Jonsson

**Affiliations:** 1 Pharmetheus AB, 752 37 Uppsala, Sweden; niclas.jonsson@pharmetheus.com; 2 Mahidol Oxford Tropical Medicine Research Unit, Faculty of Tropical Medicine, Mahidol University, Bangkok 10400, Thailand; joel@tropmedres.ac; 3 Centre for Tropical Medicine and Global Health, Nuffield Department of Medicine, University of Oxford, Oxford OX3 7LG, UK; 4 Division of Clinical Pharmacology, Department of Medicine, University of Cape Town, Cape Town 7925, South Africa; karen.barnes@uct.ac.za; 5 WorldWide Antimalarial Resistance Network (WWARN) Pharmacology Scientific Working Group, University of Cape Town, Cape Town 7925, South Africa

**Keywords:** malnutrition, translation, drug development, physiologically based pharmacokinetics

## Abstract

Malnutrition in children is a global health problem, particularly in developing countries. The effects of an insufficient supply of nutrients on body composition and physiological functions may have implications for drug disposition and ultimately affect the clinical outcome in this vulnerable population. Physiologically-based pharmacokinetic (PBPK) modeling can be used to predict the effect of malnutrition as it links physiological changes to pharmacokinetic (PK) consequences. However, the absence of detailed information on body composition and the limited availability of controlled clinical trials in malnourished children complicates the establishment and evaluation of a generic PBPK model in this population. In this manuscript we describe the creation of physiologically-based bridge to a malnourished pediatric population, by combining information on (a) the differences in body composition between healthy and malnourished adults and (b) the differences in physiology between healthy adults and children. Model performance was confirmed using clinical reference data. This study presents a physiologically-based translational framework for prediction of drug disposition in malnourished children. The model is readily applicable for dose recommendation strategies to address the urgent medicinal needs of this vulnerable population.

## 1. Introduction

Malnutrition, as in undernutrition, is a major public-health problem throughout the developing world and is an underlying factor in over 50% of the 10–11 million children under 5 years of age who die of preventable causes each year [1]. The implications of nutrient deficiency on physiology are dependent on factors such as severity, time frame, and occurrence related to age. Accordingly, different malnutrition classifications have been adopted and the most commonly used terms are stunting and wasting [2]. Stunting is caused by long-term insufficient nutrient intake and/or frequent infections, typically occurring before the age of two. Stunting is defined by a low height-for-age (HFA) and is related to adverse health and development effects, which are largely irreversible. Wasting is related to a low body weight (BWT) for height (HT) ratio, and is commonly the result of acute food shortage and/or disease [2]. Different systems and ranges for the classification of malnutrition have been adopted over the decades; among these systems, the prevalence ranges to classify levels of wasting and stunting for children below 5 years have recently been revisited [3]. The medical needs in this pediatric population are high, as the state of malnutrition is related to a high incidence of severe pathological conditions, which are either a direct consequence of the nutritional deficiency or an indirect effect of immune compromise leading to an increased prevalence of infectious diseases [1]. However, uncertainties in how and to what extent pathophysiological changes influence drug disposition prevent standard age- or weight-based dosing strategies to be defined for this patient population [4,5]. Clinical trials to identify dosing regimens for this specific patient population are very sparse, and malnourished children are often explicitly excluded from clinical trials, most likely due to the risk-adverse nature of drug development. Consequently, easily-adopted and cost-efficient methodologies that can be applied to guide urgent clinical decisions with little prior information is needed to maximize chances of successful treatment of this vulnerable population.

The importance of pediatric medication safety and efficacy has been gaining increasing attention over the past decade with new regulatory demands from authorities in USA and EU [6,7]. Although pediatric doses often have been based on allometric scaling of adult doses, the application of physiologically based pharmacokinetic (PBPK) models has proven its usefulness in designing and optimizing clinical trial designs since infants and children, in many cases, do not exhibit pharmacokinetic (PK) properties of “little adults” [8,9,10,11]. With the possibility to inform the model with prior knowledge of age-dependent changes to the organism, the PBPK modeling approach has successfully been used to establish age-dependent drug exposure relationships. This has facilitated for more efficient establishment of pediatric dosing strategies as clinical studies to determine pediatric safety and efficacy could be optimized [12,13].

The effects of malnutrition on physiology are, like age-dependent factors, more complex than just a simple reduction in total body mass. As for modeling of age-dependencies, the PBPK approach provides a possibility to account for the multi-scale changes in anatomical and physiological parameters due to different types and severity of malnutrition. This allows for a knowledge-based translation of measured consequences of malnutrition on organ volumes, body composition, and hematology. However, detailed information on the differences in body composition of malnourished children compared to a non-malnourished reference population is very limited. This complicates the development of systemic PBPK model parameters in this vulnerable population. A further complication is related to the limited availability of controlled clinical trials in malnourished children with sufficient information that could potentially be used as reference studies for model evaluation. Additionally, the majority of published clinical trials of relevance report demographic and physiological data without sufficient granularity for model evaluation, e.g., measurements are aggregated across age groups, or are not directly translatable for traditional PBPK parameterization, e.g., mid-upper arm circumference (MUAC).

This study aims to overcome these obstacles by creating a physiologically-based bridge to a malnourished pediatric population by combining information on a) the differences in body composition between healthy and malnourished adults and b) the differences between healthy adults and healthy children. This bridge would allow for predictions of drug exposure in malnourished children based on PK data in healthy adults. Furthermore, this could provide a cost-efficient method to address an urgent unmet medical need by using quantitative re-purposing of existing data and knowledge. This study presents the development and evaluation of a physiologically-based framework for prospective simulations of drug exposure in malnourished children; this framework is informed by previous knowledge on physiology of non-malnourished children and physiological consequences of malnutrition.

## 2. Materials and Methods

### 2.1. Software

PBPK models were developed using PK-Sim^®^ modeling software (Open Systems Pharmacology Suite 8.0, https://www.open-systems-pharmacology.org). Published clinical study data were digitized with WebPlotDigitizer Version 4.3 (https://automeris.io/WebPlotDigitizer/ (© A. Rohatgi)). Model input parameter optimization (Monte-Carlo) and sensitivity analysis were performed in PK-Sim^®^. The software R 3.5.3 (The R Foundation for Statistical Computing, Vienna, Austria) and RStudio 1.2.5019 (RStudio, Inc., Boston, MA, USA) were used for statistical calculation and creation of graphical data plots. PK parameters were either acquired from PK-Sim^®^ or calculated in R using the ncappc R-package.

### 2.2. Physiologically Based Pharmacokinetics

A generic whole-body PBPK model implemented in PK-Sim^®^ was adopted, including 15 organs or tissues, connected by the circulating blood system and defined by tissue volume, composition, and blood flow. Each organ consists of four sub-compartments: plasma, red blood cells, extracellular space, and intracellular space [14]. Description of tissue:plasma partition coefficient (Kp) could be calculated according to five different models, PK-Sim^®^ Standard, Rodgers and Rowland, Schmitt, Poulin and Theil, and Berezhkovskiy. The absorption model in PK-Sim^®^ divides the gastrointestinal tract into 12 segments defined by tissue volume, transit time, and pH. Each segment consists of lumen, mucosal tissue, and nonmucosal tissue. For oral solutions, the lumen of each segment is modeled as two compartments, representing the drug in solution and the fluid volume available in the luminal segment [15]. For solid oral formulations, an additional compartment connected to the dissolved drug is included [16]. Solid formulations can be described either by a release profile or by dissolution kinetics, according to the Noyes-Whitney equation describing the dissolution of spherical particles [17].

### 2.3. Modeling Physiological Changes due to Malnutrition

#### 2.3.1. Creation of Virtual Malnourished Pediatric Populations

**Figure 1:**
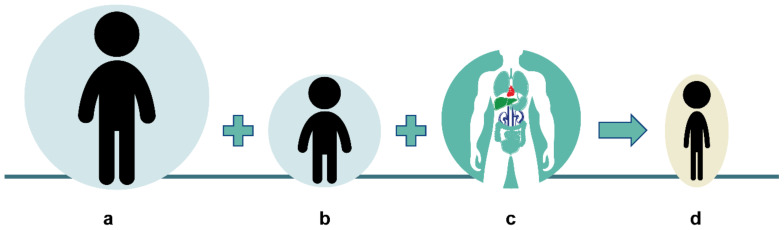
Schematic overview of the physiologically-based strategy for prediction of drug exposure in a malnourished pediatric population. The strategy involves (**a**) establishment of PBPK model for adult PK of target drug, (**b**) physiological model for pediatric scaling and (**c**) physiological model for scaling to malnutrition to enable (**d**) PK simulations of target drug in malnourished pediatric subjects.

The strategy applied for the creation of virtual malnourished pediatric populations can be summarized into two consecutive steps (Figure 1). With step one, a non-malnourished virtual pediatric population is created utilizing the in-built population algorithm in PK-Sim^®^. This functionality accounts for age-dependencies in physiology as well as maturation of biological systems, e.g., metabolic enzymes and plasma proteins. With this functionality, virtual individuals are stochastically generated applying a defined age-dependent population variability within the specified age range for the specific population. The biometric variability of these virtual populations was then reduced by excluding individuals deviating more than one standard deviation in BWT/HT from the population mean. Further details on the population algorithm have been published previously [17]. With the second step, the non-malnourished population was transformed into a malnourished population. This was accomplished by developing a set of physiological scaling parameters (PSPs), representing the physiological derangement caused by malnutrition, for the system parameters defining the whole-body PBPK model. In summary, the malnourished virtual pediatric population was created by scaling the system parameters of each virtual individual in the non-malnourished population to the developed set of PSPs. Using this approach, virtual malnourished pediatric populations can be created for any age range.

#### 2.3.2. Creation of Physiological Scaling Parameters

The literature was searched for reports including data suitable for the construction of PSPs. In this sense, reliable information is hard to obtain as physiological and morphological measures are only relevant if given as relative changes to normal conditions or if accompanied by a reference value for normal conditions. Other conditions for data to be used in a PBPK model, are that reported data should be translatable and reasonably complete, i.e., provide multiple physiological measures of malnutrition-induced changes. No suitable data set for malnourished children was found in the literature. Nevertheless, two studies on adult malnutrition were found to be appropriate, fulfilling reliability criteria as well as overcoming the abovementioned limitations, and were thus selected to be used in this study. We deemed the combined information from these studies sufficient and suitable for the intended purpose, i.e., the creation of scaling parameters describing the relative changes in malnourished physiology compared to a non-malnourished population. The selected reports included data from non-malnourished and malnourished individuals of Colombian (males, *n* = 49) and Dutch origin (males and females, *n* = 39) [18,19]. In addition to general biometrics, e.g., age, BWT and HT, these studies also included measures on hematological variables, tissue composition, and organ volumes. Furthermore, the available data allowed for calculations of PSPs at different degrees of malnutrition: mild, intermediate, and severe [18]. The data used for the generation of PSPs are summarized in Table 1. Organ and tissue PSPs were calculated as the ratio of reported malnourished and non-malnourished measurements, unless stated otherwise. Parameters describing tissue composition used to calculate specific Kp values, e.g., tissue volume content fractions, were not included in the set of PSPs. The theoretical differences in organ volumes, given the HT differences in study populations, were accounted for using typical values extrapolated from the International Commission on Radiological Protection (ICRP) database (Equation (1)):

(1) PSP=Xr,MXt,MXr,NXt,N

where *X_r_* is the reported tissue/organ value and *X_t_* is the typical ICRP tissue/organ value given the HT of study populations. *M* and *N* indicate nutritional status (*M* = malnourished, *N* = non-malnourished).

The typical values for a 30-year-old man (with HT = 176 cm and BWT = 73 kg) extrapolated from the ICRP population were used as non-malnourished reference values, unless stated otherwise. Linear extrapolations based on adjusted BWT/HT ratios were performed when information was missing for certain degrees of malnutrition.

Hematological PSPs were calculated from data reported by Barac-Nieto and colleagues [18]. The value of 4.25 g/100 mL was considered as the reference albumin concentration in non-malnourished male adults and was used to calculate the PSP for serum albumin [20]. Equal effect of nutrition status on alpha-1-acid glycoprotein levels as for albumin was assumed. The volumes of venous, arterial, and portal blood compartments were calculated using the PSP calculated for total blood volume [18].

Organ-specific measurements at conditions of non-malnutrition and severe malnutrition were available for the calculation of PSPs for brain, heart, liver, spleen, and kidneys [19]. Bone PSP was calculated based on reported bone mineral content, whereas measures of lean body mass were used as surrogates for calculation of the PSP for gonads, intestines, lung, and stomach. Skin PSP was determined based on the difference in calculated body surface area (BSA), i.e., as a function of total BWT according to the Du Bois—Du Bois formula [21]: BSA = 0.007184 × BWT^0.425^ × HT^0.725^.

Fat and muscle mass PSPs were calculated based on data reported by Barac-Nieto and Bosy-Westphal, respectively [18,19]. To ensure that the sum of organ weights added up to the target total BWT, when applying the PSPs calculated for other tissues/organs, these two PSPs were adjusted while still maintaining the relationship between these two tissues in terms of weight.

In summary, this PSP strategy enabled scaling of the system parameters while maintaining the continuity of physiological integrity and plausibility. These scaled parameters were then used to define the physiology in the established whole-body PBPK model structure implemented in the software PK-Sim^®^.

#### 2.3.3. Evaluation of Physiological Scaling

The suitability of the adopted scaling strategy and the developed PSPs was evaluated by comparing how scaling of a non-malnourished pediatric population to a malnourished pediatric population corresponded to clinical classifications of pediatric malnutrition. This check was performed both versus the World Health Organization (WHO) standards of z-score and versus a set of clinical observations [22,23]. Briefly, a z-score is calculated as (observed value—median value of the reference population)/standard deviation of the reference population. The observed reference population had previously been enrolled in an intervention study to determine the efficacy and pharmacokinetic/pharmacodynamic (PK-PD) properties of lumefantrine in African children with severe acute malnutrition (*n* = 131) and a control group of non-malnourished children (*n* = 266) [23]. This population included Malian and Nigerian children, aged 6–59 months, with uncomplicated *P. falciparum* malaria. Children were classified as having severe acute malnutrition according to the WHO criteria, i.e., weight-for-height z-score (WHZ) < −3 and/or MUAC < 115 mm. Children with kwashiorkor, severe stunting (severe chronic malnutrition given by a HFA z-score < −3), severe anemia, known underlying or chronic diseases, or other complications requiring hospitalization were excluded from the study.

The evaluation versus standard WHZ was performed by comparing the BWT/HT relationship of a virtual pediatric ICRP population of mixed sex (50:50), aged 46–48 months, considered both before and after scaling, according to the set strategy of adopting PSPs for severe malnutrition. To evaluate the PSPs versus clinical observations, we generated a virtual population from the ICRP database matching the study population in terms of age range and sex distribution. Both the non-malnourished (after removal of outliers) and scaled populations (with mild, intermediate, or severe malnutrition) were compared to the study population in terms of weight-for-age z-score (WAZ) and WHZ.

Additionally, we performed simulations to facilitate direct evaluation of the PSPs effect on simulated pediatric PK profiles. For each model drug included, simulations were performed using a representative study setup given the pediatric reference studies, e.g., population age and dose regimen. These simulations were conducted using both a non-malnourished and a severely malnourished population, generated from the non-malnourished population via the PSP translation strategy, and alike in all other aspects. This approach ensured that potential differences between simulation output, when adopting a non-malnourished or a severely malnourished virtual population, were a direct consequence of the PSPs translation. Furthermore, simulations were performed using both a BWT-adjusted and a flat dose to facilitate assessments of dose effects as well as to compare these dosing strategies.

### 2.4. PBPK Drug Models and Study Data Used in PK Evaluation

#### 2.4.1. Caffeine

The caffeine PBPK model used in this study was adapted from the one available for caffeine included in the PK-Sim^®^ model compound library. Some model refinements were performed to match the clinical plasma concentrations in healthy Nigerian adult volunteers (*n* = 10) after a 300-mg oral dose dissolved in water [24]. 

The PK evaluation of the PSP strategy was performed using extracted plasma concentration data from malnourished Nigerian children (*n* = 7) with kwashiorkor with an average age of 2.6 years (range 1.3–4.5 years) [25]. Caffeine plasma concentrations were reported after a 40-mg dose (3.6–5.6 mg/kg) administered via nasal gastric tube. Simulations were performed for a severely malnourished pediatric population replicating the reported study in terms of study population and design. The simulations were performed at post-prandial state after intake of 100 mL of a simulated nutritional drink (caloric content = 75 kcal). Evaluations were performed towards the prediction of severe malnutrition. Oral doses of 40 mg and 3.2 mg/kg, in a population with average age of 2.6 years (range 1.3–4.5 years), were used to perform simulations for direct evaluation of dose effects and dosing strategy.

#### 2.4.2. Cefoxitin

A cefoxitin PBPK model was developed to serve the purpose of this study. Initial model setup was done based on information on cefoxitin physico-chemical properties, as well as its absorption, distribution, metabolism, and excretion (ADME) properties. The PBPK model was then optimized, by parameter identification, towards the clinical plasma concentrations reported in healthy volunteers after intravenous cefoxitin doses of 2 g (5-min infusion) or 30 mg/kg (3-min infusion) [26,27]. PK evaluation of the PSP strategy was performed using reported serum concentration data after a 40-mg/kg intravenous cefoxitin dose in malnourished South African children (*n* = 6) with kwashiorkor with average age 28 ± 12 months [28]. Simulations were performed for a severely malnourished pediatric population replicating the reported study in terms of study population and design. Intravenous doses of 520 mg and 40 mg/kg, in a population with average age of 28 ± 12 months, were used to perform simulations for direct evaluation of dose effects and dosing strategy.

#### 2.4.3. Ciprofloxacin

The ciprofloxacin PBPK model used in this study was adapted from a previously published model [29]. Some model refinements were performed to match the clinical plasma concentrations in healthy American adult volunteers (*n* = 14) after a 750-mg oral dose [30]. PK evaluation of the PSP strategy was performed using extracted plasma concentration data from Kenyan children (*n* = unknown) defined as malnourished by the following criteria: WHZ ≤ 3, MUAC < 11 cm, or the presence of bilateral pedal oedema (kwashiorkor) [31]. The reported data were categorized according to age groups of 0.5, 1, 2, 5, and 10 years and ciprofloxacin plasma concentrations were reported after an oral dose of 10 mg/kg [32]. Simulations were performed for a severely malnourished pediatric population replicating the reported study in terms of study population age groups (±10%) and design. The simulations were performed at post-prandial state after intake of 200 mL of a simulated nutritional drink (caloric content = 300 kcal). Oral doses of 120 mg and 10 mg/kg, in a population with average age of 2 years (range 1.8–2.2 years), were used to perform simulations for direct evaluation of dose effects and dosing strategy.

#### 2.4.4. Lumefantrine

A lumefantrine PBPK model was developed to serve the purpose of this study. Initial model setup was based on information on lumefantrine physico-chemical, biopharmaceutical, and ADME properties. Model development was performed to include previous clinical observations on effects of food intake and susceptibility to CYP3A4 inhibition. We characterized lumefantrine intestinal absorption using data reported by a food-effect study in Chinese healthy adult volunteers (*n* = 16) as well as previous assessments of fraction absorbed (f_abs_), reported to be 4.7% and 75% in fasted and fed state, respectively [33,34]. Elimination pathway confirmation, via CYP3A4, was performed using clinical data reported by a drug-drug interaction trial with ketoconazole in Caucasian healthy adult volunteers (*n* = 16) [35]. The PK evaluation of the PSP strategy was performed versus observations of lumefantrine in malnourished Malian and Nigerien children (*n* = 131), aged 6‒59 months, with uncomplicated *P. falciparum* malaria [23]. Children were classified as having severe malnutrition according to the following criteria: WHZ < −3 and/or MUAC < 115 mm. Children with kwashiorkor, severe stunting (severe chronic malnutrition, HFA z-score < −3), severe anemia, known underlying or chronic diseases, or other complications requiring hospitalization were excluded from the study. Lumefantrine plasma concentrations were reported for a standard three-day oral twice-daily dosing of 120 mg (~15.5 mg/kg) lumefantrine given in a fixed dose combination with artemether. Simulations were performed for a severely malnourished pediatric population replicating the reported study in terms of study population and design. The simulations were performed at post-prandial state after intake of 100 mL of a simulated nutritional drink (caloric content = 75 kcal) accounting for the reported effects on bioavailability [36]. A single oral dose of 120 mg and 12 mg/kg, in a population with average age of 2.75 years (range 0.5–5 years), were used to perform simulations for direct evaluation of dose effects and dosing strategy.

#### 2.4.5. Pyrimethamine

A pyrimethamine PBPK model was developed to serve the purpose of this study. Initial model setup was done based on information on pyrimethamine physico-chemical, biopharmaceutical, and ADME properties. Model development was then performed, by parameter identification, towards: (a) clinical plasma and whole-blood concentrations reported in healthy volunteers (*n* = 7) after a 25-mg oral dose of pyrimethamine; and (b) whole-blood concentrations reported in malaria-infected adult volunteers (*n* = 228) after a 75-mg oral dose [37,38,39,40]. PK evaluation of the PSP strategy was performed using compiled observations of pyrimethamine in malnourished malaria-infected African children (n = 60) aged 16–60 months [38,39]. Children were classified as malnourished according to the criteria of WAZ < −2. Pyrimethamine whole-blood concentrations were reported after a single oral dose of 12.5, 18.75, 25, or 50 mg. Simulations were performed for a severely malnourished pediatric population replicating the reported study in terms of study population and design. Oral doses of 25 mg and 1.8 mg/kg, in a population with average age of 2.75 years (range 0.5–5 years), were used to perform simulations for direct evaluation of dose effects and dosing strategy.

#### 2.4.6. Sulfadoxine

A sulfadoxine PBPK model was developed to serve the purpose of this study. Initial model setup was done based on information on sulfadoxine physico-chemical, biopharmaceutical, and ADME properties. Model development was then performed, by parameter identification, towards: (a) clinical plasma and whole-blood concentrations reported in healthy volunteers (*n* = 7) after a 500-mg oral dose of sulfadoxine; and (b) whole-blood concentrations in malaria-infected adult volunteers (*n* = 228) after a 1500-mg oral dose [37,38,39,40]. PK evaluation of the PSP strategy was performed using compiled observations of sulfadoxine in malnourished African children (*n* = 59), aged 17–60 months, with *P. falciparum* malaria. Children were classified as malnourished according to the criteria of WAZ < −2. Whole-blood concentrations were reported after a single oral dose of 250, 375, 500, or 1000 mg of sulfadoxine. Simulations were performed for a severely malnourished pediatric population replicating the reported study in terms of study population and design. Oral doses of 500 mg and 36 mg/kg, in a population with average age of 2.75 years (range 0.5–5 years), were used to perform simulations for direct evaluation of dose effects and dosing strategy.

## 3. Results

### 3.1. Physiological Scaling Parameters

**Figure 2:**
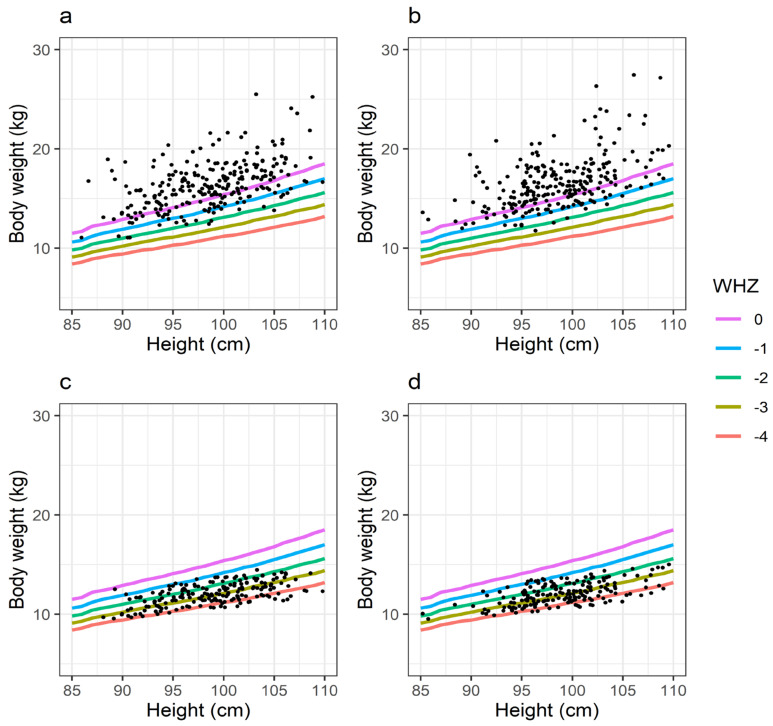
Individual body weight/height (BWT/HT) ratios for a male and female virtual pediatric population, aged 46–48 months, compared to the BWT/HT ratios according to standard weight-for-height z-scores (WHZ) before and after scaling, adopting PSPs for severe malnutrition. The lines show standard WHZ scores ranging between 0 and −4 with while dots indicate individual BWT/HT ratios. Before scaling, each population consisted of 250 individuals; after scaling and exclusion of individuals deviating more than one standard deviation in BWT/HT from the population mean, the male and female populations included 184 and 186 virtual individuals, respectively. (**a**) male population before scaling, (**b**) female population before scaling, (**c**) male population after scaling to severe malnutrition, (**d**) female population after scaling to severe malnutrition.

The calculated set of PSPs for three different levels of malnutrition (mild, intermediate, and severe) are summarized in Table 2. The largest relative change in physiology due to malnutrition was predicted to occur for plasma protein levels (PSP = 0.494), followed by organ and tissue volume of spleen (PSP = 0.612) and fat (PSP = 0.624). Consequences of scaling according to the PSPs, for different levels of malnutrition, on summary biometric parameters are visualized for a typical 30-year-old European man in Table 3 [41]. The simulated loss in total BWT was according to the reported values for malnutrition, i.e., mild (11%), intermediate (17%), and severe (26%). When applying the PSP translation strategy to a virtual pediatric population (ICRP population of mixed sex (50:50), aged 46–48 months), approximately 26% of the population were discarded as outliers, with no difference between sexes. In Figure 2, the individual BWT/HT ratios before (*n* = 500) and after scaling (*n* = 371) when adopting PSPs for severe malnutrition are shown and compared to the BWT/HT ratios according to standard WHZ. The simulated WHZ for a severely malnourished population was, on average, −3, with individual measures ranging between −1 and −4.5.

**Figure 3:**
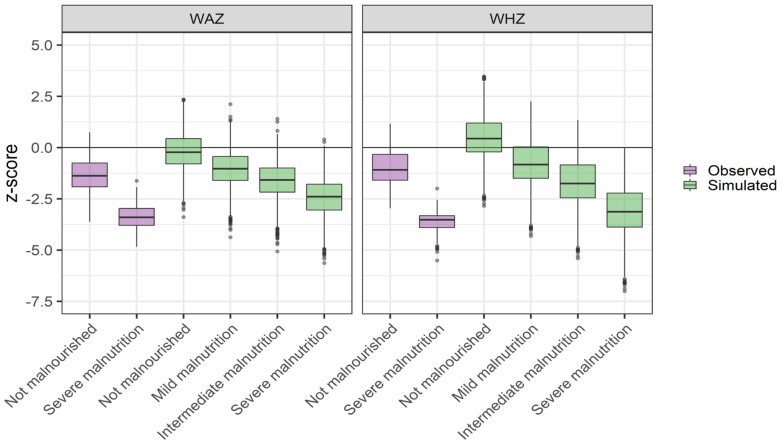
Comparison the observed weight-for-age z-scores (WAZ) and weight-for-height z-scores (WHZ) in African children, classified as either not malnourished or severely malnourished, and a virtual population at different levels of simulated malnutrition, according to the PSP translation strategy. Box plots represent median and interquartile range.

**Figure 4:**
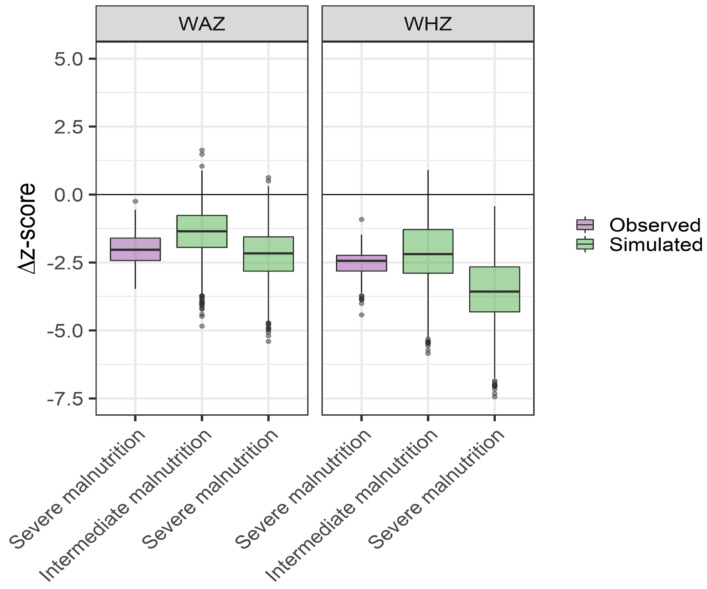
Difference in weight-for-age z-scores (WAZ) and weight-for-height z-scores (WHZ) (malnourished—not malnourished) in African children, classified as either not malnourished or severely malnourished, and a virtual population at simulated intermediate and severe malnutrition, according to the PSP translation strategy. Box plots represent median and interquartile range.

Comparisons of the WHZ and WAZ scores between a virtual population at different levels of malnutrition and observations are displayed in Figure 3 and Figure 4. Quantitative differences between simulated and observed populations was observed. Overall, the z-scores for the observed population were lower than those for the simulated one, both in the non-malnourished and severely malnourished groups (Figure 3). However, the observed and simulated populations were similar in terms of z-score differences between malnourished and non-malnourished populations (Figure 4). Based on the combined assessment of these results, severe malnutrition was selected for the PK evaluation of the scaling strategy.

### 3.2. PBPK Drug Model Development

#### 3.2.1. Caffeine

**Figure 5:**
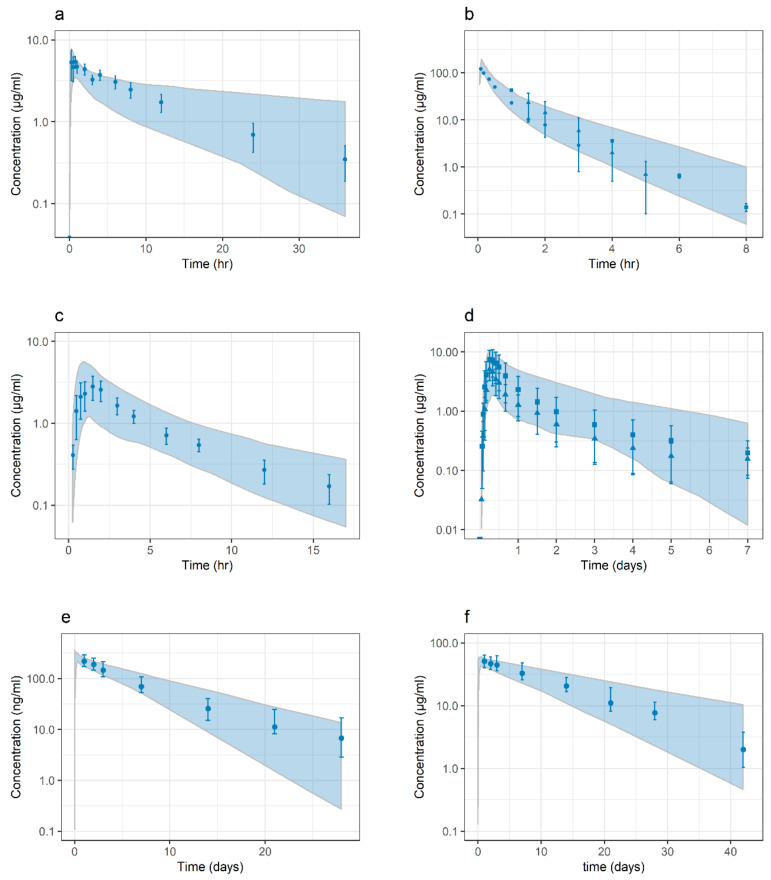
Concentration-time profiles (log-linear scale) in adult populations for (**a**) caffeine, (**b**) cefoxitin, (**c**) ciprofloxacin, (**d**) lumefantrine, (**e**) pyrimethamine and (**f**) sulfadoxine. Clinical observations are represented by dots (mean ± standard deviation [SD]). Shaded areas represent the simulated 5–95% quantiles for virtual populations. Details of clinical reference data, study design, and PBPK models are further described in the respective drugs sub-section in *PBPK Drug Models and Study Data Used in PK Evaluation* (Materials and Methods) and *PBPK Drug Model Development* (Results).

Model refinement was performed for the caffeine PBPK by parameter optimization to better represent the target population. Performance of the caffeine model was improved by optimizing logP, intestinal permeability (P_int_) and capacity for CYP1A2 metabolic clearance, via the parameter maximum rate of reaction (V_max_). The model refinement was performed adopting an oral solution for caffeine administration. All other model parameters and calculations were in accordance with the PK-Sim^®^ v8 caffeine drug model library file. Final parameters for the caffeine PBPK model are summarized in Table 4 and results from a virtual trial simulation are shown in Figure 5.

#### 3.2.2. Cefoxitin

The cefoxitin PBPK model development was informed with the measured fraction unbound in plasma (f_u.p_), calculated pKa, and solubility, whereas logP and elimination, via glomerular filtration rate (GFR) and tubular secretion, were optimized towards clinical data. The final parameters for renal elimination suggest the involvement of active renal excretion, which is in line with previous clinical observations [26]. The model development was performed adopting intravenous cefoxitin administration. Kp values and cellular permeabilities were calculated adopting the methods of Rodger and Rowlands and standard PK-Sim^®^, respectively. Final parameters for the cefoxitin PBPK model are summarized in Table 4 and results from a virtual trial simulation are shown in Figure 5.

#### 3.2.3. Ciprofloxacin

The ciprofloxacin PBPK model was adopted as reported, except for that optimization was performed to enable majority of intestinal absorption to occur in the proximal parts of the intestine. This optimization was achieved by estimation of intestinal permeability (P_int_) and the effective surface area enhancement factor (SAEF) in the caecum, while SAEF values for the small intestine and other regions of the large intestine were set to default and zero, respectively. Model development simulations were performed for ciprofloxacin administered as an oral suspension (monodisperse, particle radius = 10 µm). Final parameters for the ciprofloxacin PBPK model are summarized in Table 4 and results from a virtual trial simulation are shown in Figure 5.

#### 3.2.4. Lumefantrine

The development of a lumefantrine PBPK model was informed by reported values for logP = 2.9, pKa = 9.35, and f_u.p_ = 0.001–0.003 [33,42,43]. To reach an optimal performance, logP and f_u.p_ were estimated to 3.09 and 0.0029, respectively. The plasma binding entity of lumefantrine was modelled using albumin as a surrogate to high-density lipoproteins, since this binding entity was not available in PK-Sim^®^. The observed reduction of lipid levels in malnourished children, and its potential effects on f_u.p_, was assumed to be similar to the changes in albumin levels and would therefore be captured by the suggested PSP strategy [44,45,46]. Metabolic elimination via CYP3A4 was optimized and evaluated in relation to the reported increase in exposure after concomitant administration of the CYP3A4 inhibitor ketoconazole (area under the curve, AUC_obs_ ↑ 60.8%, AUC_sim_ ↑ 61.1%) [35]. Intraluminal solubility, at fasted and fed state, were estimated as a categorical effect along with prandial state specific dissolution, described by the Weibull function (fasted state: time to 50% dissolved = 270 min, shape = 6.9; fed state: time to 50% dissolved = 217 min, shape = 1.9). The in-vivo effect of a nutritional drink on intraluminal solubility was assumed similar to the maximum effect observed after concomitant soy milk intake. This effect was estimated assuming that the 6-fold increase observed in lumefantrine plasma exposure with soy milk intake was a direct effect of an increase in the fraction absorbed due to enhanced solubility [36]. Kp values and cellular permeabilities were calculated using the standard PK-Sim^®^ method. Final parameters for the lumefantrine PBPK model are summarized in Table 4 and results from a virtual trial simulation are shown in Figure 5.

**Table 4:** Final PBPK model parameters applied for simulations of the investigated drugs.

Parameter	Caffeine ^1^	Cefoxitin	Ciprofloxacin ^2^	Lumefantrine	Pyrimethamine	Sulfadoxine
logP	0.87	0.84 ^3^	0.95	3.09 ^3^	3.14 ^3^	3.74 ^3^
f_u.p_	0.7	0.48 ^4^	0.67	0.0029 ^3^	0.095 ^5^	0.036 ^5^
Mw	194.2	427.45	331.3	528.9	248.71	310.33
pKa	0.8 B	3.58 A ^6^	6.09 A, 8.62 B	9.35 B ^7^	6.9 B ^5^	6.2 A ^5^
Solubility ^8^	21.6 @ pH = 7	0.2 @ pH = 7 ^9^	38.4 @ pH = 7	fasted: 0.0097 ^3^ @ pH = 6.5 fed—high fat: 0.18 ^3^ @ pH = 5 fed—milk: 0.05 ^3^ @ pH = 5	0.12 @ pH = 5 ^5^	0.474 @ pH = 5 ^5^
Distribution	PK-Sim	RR	PK-Sim	PK-Sim	RR ^10^	RR ^11^
P_int_	223 ^3^	0.161 ^12^	1.57 ^3, 13^	24.4 ^3^	6370 ^3^	1690 ^3^
Renal elimination	CL_spec_ = 2.46 ×10^−3^	CL_spec_ = 3.8 ^3^ + GFR	CL_spec_ = 1.61 + GFR		GFR	GFR × 0.21 ^3^
Hepatic elimination	V_max.CYP1A2_ = 73.1 ^3^ Km_.CYP1A2_ = 14.7		CL_spec.CYP1A2_ = 0.043 CL_spec_._bile_ = 0.096	CL_int.CYP3A4_ = 93.7 ^3^	CL_spec_ = 0.089 ^3^	

f_u.p_ = unbound fraction in plasma, Mw = molecular weight (g/mol), pKa = negative base−10 logarithm of the dissociation constant, A = acid and B = base, P_int_ = intestinal permeability (10^−6^ cm/min), GFR = glomerular filtration rate, V_max_ = maximum rate of reaction (pmol/min/mg protein), Km = Michaelis-Menten constant (µM), CL_spec_ = volume normalized clearance (1/min), CL_int._ = intrinsic clearance (µL/min/mg protein). ^1^ as per PK-Sim^®^ library value if not indicated otherwise, ^2^ as reported in Schlender et al., 2018 [29] if not indicated otherwise, ^3^ optimized, ^4^ Carver et al., 1989 [27], ^5^ Charman et al., 2020 [47], ^6^ ChemAxon (http://www.chemaxon.com), ^7^ Kotila et al., 2013 [42], ^8^ solubility in mg/mL at indicated pH (@ pH), ^9^ ALOGPS 2.1 (http://www.vcclab.org/lab/alogps/), ^10^ B:P optimized via V_f.proteins_ = 0.19 and V_f.lipid_ = 0 in blood cell, ^11^ B:P optimized via V_f.proteins_ = 0.06 and V_f.lipid_ = 0 in blood cell, ^12^ calculated in PK-Sim^®^, ^13^ Absorption modification Enhancement factors for Cecum = 26.24 and other regions of large intestines = 0.

#### 3.2.5. Pyrimethamine

The PBPK model for pyrimethamine was developed based on input data on f_u.p_, pKa, and solubility reported by Charman et al. in 2020 [47]. Final model parameters were obtained by optimizing logP and hepatic elimination. Furthermore, to adequately describe whole-blood concentrations, while still maintaining the possibility to translate values using the PSP strategy, the parameters that determine partitioning to erythrocytes, i.e., the V_f_ of proteins and lipids in erythrocytes, were optimized. Non-specific hepatic elimination was adopted as no information on specific metabolic pathways could be found. Renal elimination described by GFR was also included and contributed to approximately 30% of the total elimination, in accordance with previous observations [48]. Kp values and cellular permeabilities were calculated adopting the methods of Rodger and Rowlands and the standard charge-dependent Schmitt normalized to PK-Sim^®^, respectively. Model development simulations were performed for pyrimethamine administered as solid formulation, assuming dissolution kinetics as described by a Weibull function (time to 50% dissolved = 10 min, shape = 1). Final parameters for the pyrimethamine PBPK model are summarized in Table 4 and results from a virtual trial simulation are shown in Figure 5.

#### 3.2.6. Sulfadoxine

The PBPK model for sulfadoxine was developed based on input data on f_u.p_, pKa, and solubility reported by Charman et al. in 2020 [47]. Final model parameters were obtained by optimizing logP and renal elimination, via the GFR factor. Furthermore, to adequately describe whole-blood concentrations, while still maintaining the possibility to translate values using the PSP strategy, the parameters that determine partitioning to erythrocytes, i.e., the V_f_ of proteins and lipids in erythrocytes, were optimized. In agreement with previous clinical reports, the model suggests that sulfadoxine elimination is slower than that calculated using GFR, thus indicating tubular reabsorption of sulfadoxine [49]. Kp values and cellular permeabilities were calculated adopting the methods of Rodger and Rowlands and standard charge dependent Schmitt normalized to PK-Sim^®^, respectively. Model development simulations were performed for sulfadoxine administered as solid formulation, assuming dissolution kinetics as described by a Weibull function (time to 50% dissolved = 10 min, shape = 1). Final parameters for the sulfadoxine PBPK model are summarized in Table 4 and results from a virtual trial simulation are shown in Figure 5.

### 3.3. Pharmacokinetic Evaluation of Physiological Scaling Parameters

Systemic concentration-time profiles of the model drugs included in this study were simulated for severely malnourished pediatric virtual populations and then compared with clinical reference data. Reference data for caffeine and cefoxitin were only available as means with standard deviations [25,28]. Clinical data for ciprofloxacin did not allow for AUC calculations on an individual level. Hence, clinical exposure (represented by AUC) was calculated for each age group from mean profiles, and the population mean value was then calculated as mean of the AUCs from different age categories [32]. Reference exposure (AUCs) for lumefantrine, pyrimethamine, and sulfadoxine was calculated for a single dose administration based on individual co-variates and parameter estimates from previously reported population PK analyses: dose × bioavailability/CL [23,40,50].

**Figure 6:**
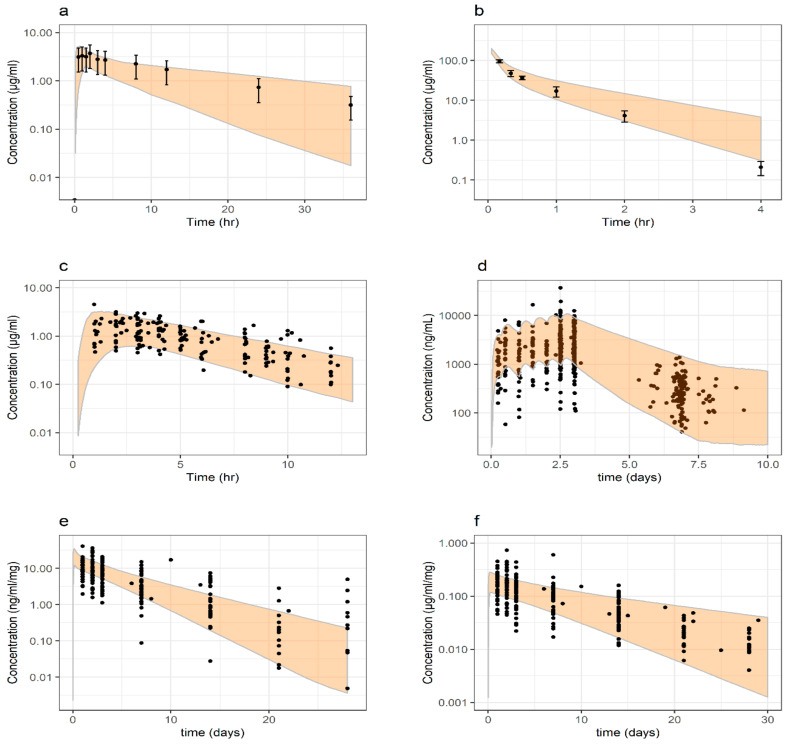
Concentration-time profiles (log-linear scale) in severely malnourished pediatric populations, (**a**) caffeine, (**b**) cefoxitin, (**c**) ciprofloxacin, (**d**) lumefantrine, (**e**) pyrimethamine and (**f**) sulfadoxine. Clinical observations (reported either as mean ± SD or individual measurements) are represented by dots. Shaded areas represent the predicted 5–95% quantiles for virtual populations. Details of clinical reference data, study design, and PBPK models are further described in the respective drugs sub-section in *PBPK Drug Models and Study Data Used in PK Evaluation* (Materials and Methods) and *PBPK Drug Model Development* (Results).

**Figure 7:**
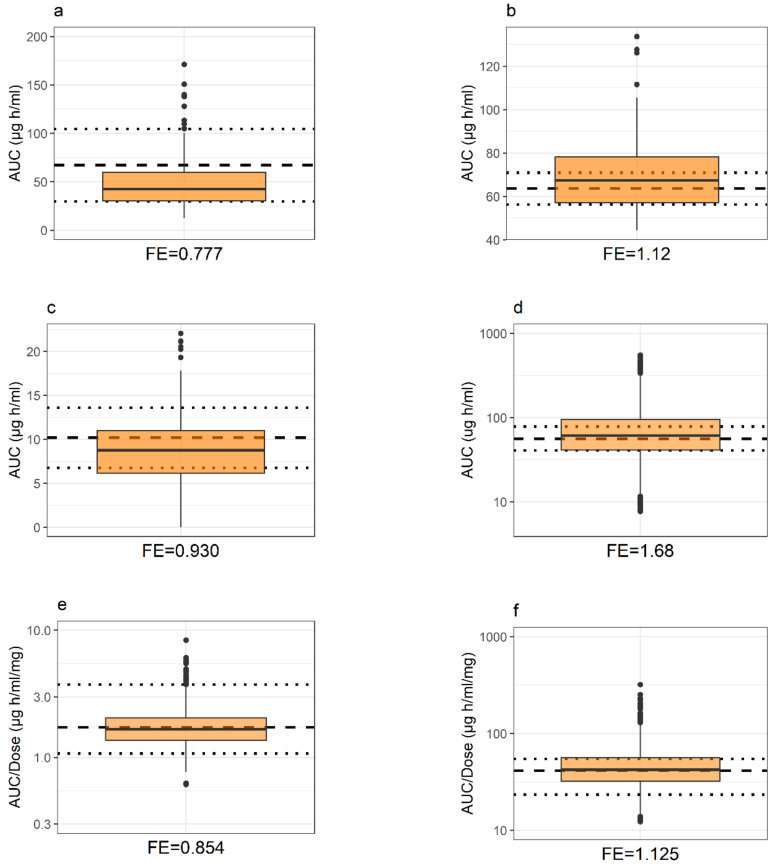
Systemic drug exposure, i.e., AUC, in severely malnourished children after a single-dose administration, (**a**) caffeine, (**b**) cefoxitin, (**c**) ciprofloxacin, (**d**) lumefantrine, (**e**) pyrimethamine and (**f**) sulfadoxine. Box plots represent predictions for a virtual population while horizontal dashed and dotted lines either indicate the observed mean ± SD (caffeine, cefoxitin, and ciprofloxacin) or the median (Q1, Q3) (lumefantrine, pyrimethamine and sulfadoxine) from clinical reference studies. Fold error (FE) is calculated as the simulated to observed ratio of specified AUC central tendency measure. Details of clinical reference data, study design, and PBPK models are further described in the respective drugs sub-section in *PBPK Drug Models and Study Data Used in PK Evaluation* (Materials and Methods) and *PBPK Drug Model Development* (Results).

Pediatric population demographics and trial design were selected to replicate clinical reference studies. PSPs representing severe malnutrition and developed PBPK models were applied accordingly. The number of individuals considered in the severely malnourished pediatric virtual populations were 82, 68, 361, 3250, 3313, and 3257 for the simulations of caffeine, cefoxitin, ciprofloxacin, lumefantrine, pyrimethamine, and sulfadoxine, respectively. Overall, simulated systemic concentration-time profiles agreed well with clinical observations (Figure 6). Similarly, the systemic exposure was predicted with adequate accuracy with a simulated-to-observed mean AUC-ratio of 0.78, 1.12, 0.93, 1.68, 0.854, and 1.13 for caffeine, cefoxitin, ciprofloxacin, lumefantrine, pyrimethamine, and sulfadoxine, respectively (Figure 7). The absolute average deviation of simulations to observations in AUC was 1.22-fold, while the average error was 1.08-fold. Predicted population variability in systemic PK was similar to observations for caffeine, cefoxitin, lumefantrine, and sulfadoxine, while it was to some extent underpredicted for pyrimethamine and sulfadoxine.

**Figure 8:**
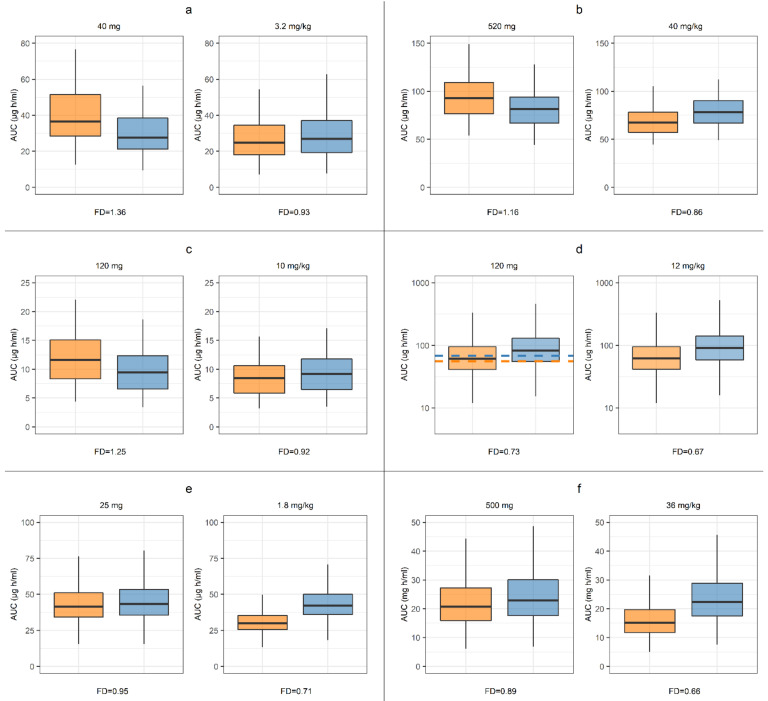
Predicted systemic drug exposure in severely malnourished (orange) or non-malnourished (blue) children after single-dose administration, (**a**) caffeine, (**b**) cefoxitin, (**c**) ciprofloxacin, (**d**) lumefantrine, (**e**) pyrimethamine and (**f**) sulfadoxine. Dotted lines included for fixed-dose administration of lumefantrine represent the observed median for the respective nutritional status. Fold deviation (FD) is calculated as the median AUC ratio between severely malnourished and non-malnourished. Box plots represent median and interquartile range. Simulations were performed for a flat dose (mg) and per body weight (mg/kg), and according to reference simulations performed in the *Pharmacokinetic Evaluation of Physiological Scaling Parameters* (Results). Details of study design and PBPK models are further described in the respective drugs sub-section in *PBPK Drug Models and Study Data Used in PK Evaluation* (Materials and Methods) and *PBPK Drug Model Development* (Results).

The simulated effects of severe malnutrition and the implication of flat or BWT-adjusted dose on the exposure to the drugs included are illustrated in Figure 8. According to the simulations using a BWT-adjusted dose, a reduction in simulated exposure is to be expected for all drugs. Notably, our simulations led to substantial differences in the level of these reductions, ranging from AUC_malnourished_/AUC_not-malnourished_ ratios of 0.66 (sulfadoxine) to 0.93 (caffeine). Similarly, the simulated effects of malnutrition on exposure when adopting a flat-dose regimen (i.e., dose set according to age) showed large differences among the investigated drugs. The relative exposure (AUC_malnourished_/AUC_not-malnourished_) at malnutrition was predicted to be higher for caffeine (1.36), cefoxitine (1.16), and ciprofloxacin (1.25), lower for lumefantrine (0.73) and sulfadoxine (0.89), and remain similar for pyrimethamine (0.95). Clinical observations for the flat-dose regimen of lumefantrine were also available for a non-malnourished population, which confirmed the simulation results.

## 4. Discussion

PBPK modeling simulates PK profiles on the basis of compound-related information and a model structure parameterized with relevant physiological input parameters of the individual, such as organ volumes, tissue composition, blood flow rates, and clearance. In essence, the model structure and parametrization, i.e., the system, of a PBPK model aims to describe the organism. The traditional way to accomplish this is to inform the model with as detailed information as possible on the anatomical and physiological characteristics of the target population. Once the model has been established, the appropriateness of the model should be verified to ensure that representative virtual populations can be generated and that these are suitable for the intended purpose, i.e., PK simulations. The aim of this study was to establish a generic PBPK framework for simulations and predictions of PK properties in malnourished children. To this aim, we needed to define and verify the alterations to physiology induced by malnutrition. In literature, quantitative anthropometric measurements (HT, BWT, body mass index, skinfold thickness, and MUAC) of malnourished children are abundant. However, since these measurements do not provide any information at a tissue or organ-level, they have little value for the purpose of informing PBPK models. Well-defined reference values at normal conditions, i.e., normal nutritional status, are also often missing. In the absence of target population data, an alternative approach was adopted to reach the set study goals. Under the assumption that in a state of malnutrition similar physiological alterations occur for children and adults, a physiologically-based bridge to a malnourished pediatric population was developed. This was achieved by combining information on a) the differences in body composition between non-malnourished and malnourished adults and b) the differences between adults and healthy children in a normal nutritional state. A set of PSPs to scale a non-malnourished population to different levels of malnutrition was established based on previously published measurements and the physiological database included in the PBPK platform PK-Sim^®^. By this strategy, virtual populations representing different target malnourished pediatric patient populations were generated, and accurate PK predictions were achieved. However, it should be noted that the current approach presents a generic translation of body composition and does not account for potential changes to specific physiological attributes and functionalities, such as specific tissue composition and capillary fenestration, which may be important for the disposition of certain drugs. Consequently, although the results support the overall appropriateness of the presented strategy, specific interpretations should be made with care. Finally, as the presented strategy was adapted to be used with the open access and open source software PBPK platform PK-Sim^®^, it is available for any organization to be used and further developed [51].

The final PSPs included malnutrition-induced alterations to tissues, organs, and plasma protein levels as defined in the PBPK model structure in PK-Sim^®^ (Table 2). To accomplish this, data from two different studies were used [18,19]. Even though relative changes compared to a normal nutritional state were established in both publications, inter-study bias may have been introduced when combining the collated information. The PSPs for severe malnutrition simulated a 26% loss in BWT with different levels of effect on organs/tissues. A higher relative loss was simulated for fat (38%), kidney (31%), liver (32%), muscles (29%), and spleen (39%). As previously discussed, absolute comparisons between studies are hard to perform, but our estimated effects of PSPs, e.g., on BWT reduction and specifically affected organs, are in agreement with previous reports [52]. One important note is that malnutrition is a heterogeneous condition in terms of manifestations, e.g., marasmus and kwashiorkor are two conditions that have not been accounted for in this study. It should also be noted that the state of “severe malnutrition” reported in this study does not represent lethal malnutrition, i.e., lethal starvation, which occurs at an approximate 40% loss of BWT. These conditions may have additional implications for drug disposition [4]. However, the innate difficulty to study drug disposition in children with such life-threatening malnutrition is further complicated by many confounding variables caused as a results of concomitant malnutrition management and the prevalence of comorbidities [53]. Furthermore, we propose no distinction in applicability of the suggested strategy related to the malnutrition classes stunting and wasting. The rationale for this is that there are little evidence supporting a significant difference between these categories in terms of changes in tissue and organ weights compared to a non-malnourished state, which is the basis of the PBPK modeling approach. In this sense we therefore consider that the proposed strategy will work equally well for both categories and that it will be helpful, rather than harmful, for both classes. This is also supported by that adequate predictions were acquired overall, although the patient populations were in different states of malnutrition, including kwashiorkor. As knowledge increase, both regarding data and information but also by further evaluation, refinements to the strategy may be achieved.

The use of PSPs also indirectly influences the virtual cardiac output (CO), i.e., the sum of blood flows in each organ/tissue excluding lungs, as blood perfusion in each organ/tissue is parameterized as blood flow per organ/tissue weight. Consequently, when applying the PSPs, the absolute perfusion rate will decrease linearly with the reduction in organ weight. Hence, the simulated effect of malnutrition on CO will be a function of absolute reduction of tissue/organ weights, specific blood flow per organ/tissue weight, and the relative mass of each organ/tissue. In average, the simulated change in CO according to the PSPs is approximately 23%. The implications of this effect in relation to the observations are hard to assess. For instance, although the CO was reported to decrease by 48% after 6 months of semi-starvation, the observed circulatory index (i.e., cardiac function related to metabolic demand) was unchanged [52].

The presented set of PSPs does not include parameters related to GI functionality even though some changes in oral, gastric, and small intestinal physiology have been reported [54]. The co-prevalence of GI diseases in populations suffering of malnutrition leads to an inherent difficulty to distinguish the effects of malnutrition on GI physiology and function [52]. Although some general trends pointing towards GI alterations due to malnutrition have been suggested, these could not be linked to clinical relevance for absorption of orally administered drugs [54]. This could be related to the fact that the capabilities of food digestion and nutrient absorption are rarely lost despite that alterations to the GI tract may occur [52]. Overall, the lack of detailed information about patients and study design, as well as the absence of data from representative reference populations, reduce the possibility to discriminate underlying mechanisms from observations. Consequently, in some cases, changes of PK profiles have bene attributed to variations in absorption while they were most likely due to changes in clearance and age-dependent first-pass effects [4,54]. Changes of PK profiles have also been interpreted as an alteration in elimination capacity [4,5]. At the same time, numerous reports state that renal function is unaffected by malnutrition and no direct evidence exists on malnutrition-induced changes in enzyme abundances for enzymes commonly involved in drug metabolism, e.g., cytochrome P450 and uridine 5′-diphospho-glucuronosyltransferase family [52,55]. A contributing element to this discrepancy may be that other factors than functionality of the eliminating organs, such as distribution and protein binding, can influence the rate of drug disappearance from the systemic circulation. This may have influenced the interpretation of effects observed in clinical studies. Nevertheless, due to the high prevalence of disease in these populations, comorbidities and disease-induced effects may need to be accounted for when performing model simulations involving such populations [52,55,56,57].

When adopting the PSPs to generate a severely malnourished virtual pediatric population, the population’s WHZ was −3, on average, and ranged between −1 and −4.5 (Figure 2). The inclusion of individuals with WHZ > −3, although they do not meet the WHO definition of severe malnutrition, may seem counterintuitive. However, the scaling strategy should be viewed from the perspective of scaling each individual in a population at a normal nutrition level to a fixed state of malnutrition. In practice, the use of PSPs will lead to an equal drop in WHZ across the population, and the WHZ of each individual at malnutrition will depend on his/her original WHZ. The drawback of this approach is, as mentioned above, that individuals with a WHZ > −3 also will be included. The benefits are that (a) direct comparisons of drug disposition in populations at different nutritional states can be made, and (b) interindividual variability in body composition, as well as integrity of physiology, are maintained. For the specific purpose of assessing the overall implications of malnutrition for drug disposition, we believe that the benefits of the approach prevail over the drawbacks. When comparing a virtual population with a target patient population, clear differences were observed with the “non-malnourished” reference population (Figure 3). This was expected given that virtual populations were created on the basis of the ICRP database, which includes detailed information on age and gender-related differences in the anatomical and physiological characteristics of reference individuals for a western European population [41]. The ICRP database was selected and used throughout this study given the lack of a specific database on the African population. Although an “African population” cannot be defined due to the great heterogeneity in Africa, some discrepancies in population biometrics, and potentially also in absolute outcome, were expected. However, the relative effect of malnutrition was anticipated to be less dependent on these factors, as we have confirmed (Figure 4). Based on these results, the simulated implications of severe malnutrition were used throughout the study. In addition, since the majority of drug PBPK models used (except those for cefoxitin and ciprofloxacin) were developed using clinical reference data collected from African adults, the potential effects that using the ICPR population may have had on PK profiles was reduced. The calibration of the PBPK models to an African population consequently also increases the appropriateness for PK predictions in African children.

The suggested approach was able to accurately predict PK profiles and parameters in severely malnourished children for six drugs with a wide range of ADME properties, such as different routes of elimination, level of protein binding, extent of tissue distribution, and potential for intestinal absorption after oral administration. This was reflected in an absolute average deviation in predicted to observed systemic exposure of 1.22-fold with no systematic trend for under- or overprediction, indicated by an average error of 1.08-fold. The diversity in drug properties supports the overall appropriateness of the suggested PSPs translation strategy for generic and prospective simulations of drug disposition in a malnourished pediatric population. Although the simulated variability in plasma exposure was well predicted overall, the available reference data did not allow further analyses to investigate the mechanisms behind the variability. The model strategy allows one to integrate, when available, additional knowledge on specific target patient populations. e.g., higher granularity in demographics or disease-related effects, to further inform the model for increased specificity and performance.

By comparing the simulated exposure of the investigated drugs after a fixed or a BWT-adjusted dose, shows that the implications of malnutrition can be expected to vary among drugs (Figure 8). For instance, the preferable dose regimen to attain comparable exposure in a severely malnourished population would be a fixed dose for pyrimethamine and a BWT-adjusted dose for caffeine (Figure 8). The difference can be related to the simulated tissue distribution, which in the PBPK model are determined by several parameters, such as f_u.p_, logP, and molecular weight. Additionally, by adopting the PBPK methodology one can account for, and further investigate, several aspects of potential relevance, such as dose regimens, routes of administration, food effects, and selection of drug delivery system. The use of this model for these patient populations also allows for traditional PBPK applications, such as drug-drug interactions and predictions of dose non-linearities due to saturation or solubility-limited absorption. The latter aspect is especially relevant for the simulation outcome of the poorly soluble drug lumefantrine, included in this study. When dosed by BWT, the bioavailability of lumefantrine will be higher in lighter subjects, and thus a higher fraction of the dose will be dissolved before intralumenal saturation occurs. In addition, since lumefantrine is eliminated via CYP3A4, which reaches full maturation at ~3 years of age, this drug provides an example of complex age and dose-dependent drug exposure that would be difficult to assess with means other than PBPK. Given these complex exposure dependencies, the accurate predictions obtained for lumefantrine further support the appropriateness of the proposed modeling strategy (Figure 7).

## 5. Conclusions

This study presents a physiologically-based translational framework for prediction of drug disposition in children with severe malnutrition by repurposing existing data and knowledge. The translational approach presented is readily applicable for dose recommendation strategies to address the urgent medicinal needs of this highly vulnerable population.

## Figures and Tables

**Table 1:** Reported physical characteristics of study populations used to calculate physiological scaling parameters (PSPs).

Study	Measurement				
	Barac-Nieto ^1^			Bosy-Westphal ^2^	
Nutritional category ^3^	M	I	S	IW	UW
Weight (kg)	52.03	48.24	42.52	70.9	46.3
Height (cm)	156	157	156	178	165
BWT/HT (kg/m)	33.3	30.8	27.4	39.8	28.1
% of standard BWT/HT	89.5	82.7	73.9		
Serum albumin (g/100 mL)	3.8	3	2.1		
Hematocrit	44.4	37.2	32		
Fat mass (%)	17.7	19.8	15.2		
Brain (kg)				1.51	1.13
Heart (kg)				0.33	0.22
Liver (kg)				1.64	0.94
Spleen (kg)				0.23	0.11
Kidney (kg)				0.36	0.21
BMC (kg)				2.68	1.93
LBM_trunk_ (kg)				24.1	16.9
MM (kg)				27.6	16.6

BWT = body weight, HT = height, M = mild nutritional impairment, I = intermediate nutritional impairment, S = severe nutritional impairment, IW = intermediate weight, UW = underweight, BMC = bone mineral content, LBM_trunk_ = Lean soft tissue trunk, MM = skeletal muscle mass. ^1^ Barac-Nieto et al. [18]. ^2^ Bosy-Westphal et al. [19]. ^3^ as defined in publications.

**Table 2:** Derived physiological scaling parameters for translation of physiological changes at different levels of malnutrition.

Component		Not Malnourished	Malnutrition Level		
			Mild	Intermediate	Severe
Bone		1	0.947	0.913	0.869
Brain		1	0.918	0.865	0.797
Fat		1	0.817	0.822	0.624
Gonads, intestines, lung, stomach		1	0.936	0.894	0.84
Heart		1	0.902	0.839	0.758
Kidney		1	0.874	0.792	0.686
Liver		1	0.872	0.789	0.682
Muscle		1	0.893	0.771	0.715
Pancreas		1	0.936	0.894	0.84
Skin		1	0.954	0.922	0.879
Spleen		1	0.844	0.743	0.612
	arterial	1	1.03	0.992	0.833
Blood	venous	1	1.02	0.979	0.822
	portal vein	1	1.02	0.982	0.825
Plasma proteins		1	0.894	0.706	0.494
Hematocrit		1	0.945	0.791	0.681

**Table 3:** Consequences of scaling according to the PSP strategy, for different levels of malnutrition, on summary biometric parameters for a typical 30-year-old European man [41].

Biometric	Not Malnourished	Malnutrition Level		
		Mild	Intermediate	Severe
BWT (kg)	73.0	65.3	60.3	53.9
HT (cm)	176	176	176	176
BMI (kg/m^2^)	23.6	21.1	19.5	17.4
BWT/HT (kg/m)	41.4	37.1	34.3	30.6
BSA (m^2^)	1.9	1.8	1.7	1.7

BWT = body weight, HT = height, BMI = body mass index, BSA = Body surface area.

## Data Availability

Data sharing not applicable.
